# Impact of early enteral nutrition combined with fine nursing in patients with intracranial aneurysm undergoing minimally invasive surgery

**DOI:** 10.3389/fsurg.2025.1632738

**Published:** 2026-01-06

**Authors:** Yujia Fu, Mengya Xiong, Tian Zuo, Yun Li, Ru Wang, Dongmei Wang

**Affiliations:** Department of Neurosurgery, Changzheng Hospital, Naval Medical University, Shanghai, China

**Keywords:** early enteral nutrition, fine nursing, immune function intracranial aneurysm, minimally invasive surgery, neurocritical care

## Abstract

**Background:**

Postoperative recovery in patients undergoing minimally invasive surgery for intracranial aneurysms is often hindered by delayed mobilization, inadequate nutritional support, and prolonged hospital stay. Conventional care using parenteral nutrition and standard nursing practices may not sufficiently address these issues. This study evaluated the combined effects of early enteral nutrition and enhanced nursing care on recovery outcomes.

**New method:**

A total of 138 patients were divided into a control group receiving standard nursing care and parenteral nutrition and an observation group receiving enhanced nursing care alongside early enteral nutrition. Recovery outcomes, including time to ambulation, gastrointestinal function recovery, hospital stay duration, pain levels, complication rates, nutritional and immune function, quality of life, and nursing satisfaction were assessed.

**Results:**

The observation group showed significantly earlier mobilization, faster gastrointestinal recovery, and shorter hospital stay than the control group. Pain levels and complication rates were lower, and nursing satisfaction was higher. Nutritional and immune markers and quality of life scores improved significantly in the observation group.

**Conclusions:**

Early enteral nutrition combined with enhanced nursing care improves recovery, reduces complications, and enhances overall patient satisfaction, presenting an effective alternative to conventional care for patients undergoing intracranial aneurysm surgery.

## Introduction

Intracranial aneurysm a cerebrovascular disease with a high clinical incidence, and its specific manifestations are mainly abnormal cystic bulging of intracranial arteries (1). Clinical studies have found that intense exercise, forced defecation, emotional agitation, and cough may cause intracranial aneurysm rupture, leading to subarachnoid hemorrhage, which seriously harms the safety of patients (2). Interventional embolization is one of the most commonly used methods in the clinical treatment of intracranial aneurysms. It is not only effective, but also minimally invasive, quick to recovery from, involves minimal damage, and is safe and reliable (3). However, due to the sudden onset and rapid progression of intracranial aneurysms, some patients may experience aneurysm rupture during the operation and are prone to complications such as hydrocephalus, rebleeding, and cerebrovascular spasm after surgery, which seriously affect the prognosis of patients (4). Hence, it is important to implement effective nursing measures to improve the symptoms and prognosis of patients.

The conventional nursing model focuses on the disease itself, with the completion of the treatment task as the primary goal, ignoring the mental health of the patient (5). The fine nursing intervention mode pays attention to the psychological and social needs of patients while paying attention to the physical diseases of patients and provides timely guidance to psychological disorders so that they can restore their normal life ability, along with promoting treatment compliance and treatment effect of patients (6).

In the field of neuro-intensive care, the nutritional status of patients is closely related to the treatment outcome (7). Neuro-intensive patients, especially those with critical conditions such as intracranial aneurysms, often experience metabolic disorders and insufficient nutrient intake due to the disease itself, surgical stress, and prolonged bed rest (8). Malnutrition further weakens the immune function of patients, increases the risk of infection, delays wound healing, affects the recovery of neurological functions, and ultimately leads to prolonged hospital stays, increased medical costs, and even an increased risk of death (9). Therefore, appropriate nutritional support is of vital importance for the treatment and rehabilitation of neuro-intensive patients.

Enteral nutrition, as an important method of nutritional support, plays a crucial role in the treatment of neurocritical conditions (10). Compared with parenteral nutrition, early enteral nutrition is more in line with the physiological state, which can maintain the integrity and function of the intestinal mucosa, promote intestinal peristalsis and secretion of digestive juices, and reduce the occurrence of intestinal bacterial translocation and infection (11). Numerous studies have shown that early enteral nutrition can improve the nutritional status of patients with neurocritical conditions, enhance immune function, reduce the incidence of complications, shorten the duration of mechanical ventilation and hospitalization, and has a positive impact on improving the treatment outcome of patients (12).

In this study, we aimed to explore the impact of early enteral nutrition in combination with fine nursing in patients with intracranial aneurysms undergoing minimally invasive surgery.

## Data and methods

### General data

This study was a randomized controlled study. One hundred and thirty-eight patients with intracranial aneurysms who underwent endovascular interventional embolization at our hospital between May 2022 and May 2023 were selected as the study participants. This study was conducted in accordance with the ethical principles outlined in the Declaration of Helsinki and was approved by the Ethics Committee of Changzheng Hospital, Naval Medical University. Inclusion criteria: (1) All patients met the diagnostic criteria for intracranial aneurysms and were confirmed by craniocerebral CT, MRI and digital subtraction angiography; (2) Patients and their families understood the purpose and content of the study, voluntarily participated in the study and signed the informed consent. Exclusion criteria: (1) Complicated heart, liver, kidney and other serious organ failure and blood system diseases; (2) Patients with communication disorders and severe mental illness; (3) Poor compliance, and inability to cooperate with the study; (4) Patients with a history of bleeding disorders.

### Random grouping method

Among the 138 patients who met the inclusion criteria and did not meet the exclusion criteria, they were grouped using the random number table method. The specific procedure was as follows: First, the 138 patients were sequentially numbered from 1 to 138 based on their admission order. Then, an arbitrary starting point was selected from the random number table, and three-digit random numbers were read in a certain direction (such as from left to right). For each random number, if its value was within the range of 1–138 and the corresponding number had not been selected before, the patient with that number was included in the study; if the read random number exceeded the range of 1–138 or the number had been selected, the number was skipped and the next one was read, until all 138 patients were selected. Finally, the patients were divided into the control group (CG) and the observation group (OG) based on the parity of the random numbers. Patients with odd numbers were assigned to the CG, and those with even numbers were assigned to the OG, with 69 patients in each group. No differences were observed in the general data between the two groups (*P* > 0.05, Table 1).

**Table 1:** General data of patients between 2 groups.

Items		Control group	Observation group	*Χ* ^2^	*P*
	Gender (male/female)	35/34	36/33	0.029	0.170
	Average age (years)	58.86 ± 5.46	58.83 ± 5.41	0.032	0.974
Aneurysm location	Anterior communicating aneurysms	27	28	0.146	0.985
	Middle cerebral aneurysm	11	10		
	Internal carotid artery bifurcation aneurysm	9	8		
	Posterior communicating aneurysms	22	23		
Hunt-Hess grade	Grade 0	7	8	0.401	0.939
	Grade I	26	27		
	Grade II	19	20		
	Grade III	17	14		

### Nursing methods

The CG received routine nursing interventions, including health education, diet guidance, health care, and environmental care.

The OG received fine nursing care based on the routine nursing. (1) Nurses conducted a timely head and neck examination of the patient to determine the degree of vascular lesions, placed an indwelling vein needle in the left upper limb to ensure smooth infusion, promptly understood the past medical history and the cognitive level of endovascular embolization after admission, and accurately assessed the psychological state. Nurses provided targeted psychological counselling to the patients, explained the importance and necessity of endovascular embolization treatment to the patients and their families, and informed them about the treatment methods, surgical procedures, precautions, complications, etc. Nurses strengthened health education based on unmastered knowledge to improve patients' mastery of health knowledge and encourage patients to actively cooperate with treatment. Nurses explained successful cases to eliminate patients' nervousness, fear, anxiety, and other emotions, help patients build confidence, and improve compliance with treatment and nursing.

1. Intraoperative nursing: The patient's vital signs were closely monitored during the operation. To prevent tumor rupture, blood pressure was measured every 5 min. To prevent thrombosis, heparin saline was administered intravenously at a dose of 3,000 IU, which was halved after 1 h, followed by 1,000 IU.
2. Postoperative nursing: After surgery, the nurse assisted the patient to maintain the supine position for 12 h and closely observed the skin color and temperature changes at the extremities of the operative side to prevent excessive compression and excessive compression of the femoral artery, which led to the blockage of blood flow to the lower extremities. At the same time, the nurses asked the patient to drink more water and assisted the patient to urinate as soon as possible and move the limbs appropriately. The nurse observed changes in urine volume, breathing, heart rate, blood pressure, and consciousness and dealt with any abnormalities in a timely manner.
3. Dietary nursing: The patient was instructed to refrain from eating within 6 h after surgery, to mainly drink water for 6 h after surgery, and then to eat a high-protein, high-vitamin, easy-to-digest diet, and keep stool unobstructed.
4. Puncture site nursing: The puncture site was compressed using a sandbag for 4 h after surgery and wrapped with elastic bandages for more than 24 h. Nurses regularly monitored the location of the patient's puncture site and intervened promptly to address fluid seepage, blood seepage, swelling, and other conditions to improve patient comfort.
5. Pain intervention: Nurses assisted the patient's family members in carrying out simple massage, during which the patient's whole body was relaxed, twice a day for 15 min each time.
6. Hydrocephalus nursing: Six hours after the operation, the head of the bed was raised 30° to enhance the effect of intracranial venous return, and the patient's consciousness and urination were observed. In cases of urinary incontinence or slow recovery of consciousness, head CT was reviewed in time, and dehydrating drugs were administered in cases of hydrocephalus.
7. Cerebral vasospasm: Nurses paid attention to the recovery of the patient's limb and speech disorders, observed the pupil, consciousness, and other signs, and paid attention to whether there was a new neurological dysfunction or severe pain. If the above situation occurred, nurses promptly notified the doctor.
8. Aneurysm rupture: Nurses observed patients with symptoms such as unconsciousness and sudden severe pain, timely reviewed the head CT, and instructed family members to pay attention to the patient's emotions and avoid stimulating the patient.
9. Discharge guidance: Upon discharge, the patient was asked to carry out daily care of the puncture site and pay attention to changes around the puncture site. If adverse conditions occur, the patient should visit the hospital for a follow-up consultation at the first time. The diet should be light, with small and frequent meals, avoiding irritating food, and proper exercise.

### Nutrition support methods

The CG received routine parenteral nutrition intervention: the patients were administered intravenous infusion of fat emulsion for 24 h (Yangzijiang Pharmaceutical Group Co., LTD., 50 mL: 0.5 g), amino acid injection (Guizhou Shengjitang Pharmaceutical Co., LTD., 500 mL: 25 g), and appropriate amounts of vitamins and electrolytes were added according to the patient's condition.

The OG adopted early enteral nutrition intervention through a nasogastric tube. Nutrison Fibre [Nutricia Pharmaceutical (Wuxi) Co., Ltd.] was administered at a constant rate of 20–30 mL/(kg·d) and the temperature was maintained at 37–42 ℃. The initial pumping rate was 20 mL/h, which was increased to 50 mL/h according to the patient's condition.

### Observation indicators

1. The first time of getting out of bed, gastrointestinal function recovery time and length of hospital stay were compared between the two groups.
2. The VAS scale was used to score the degree of pain in the two groups on day 1, day 3 and day 7 after surgery.
3. Hamilton Anxiety Scale (HAMA) and Hamilton Depression Scale (HAMD) were implemented for assessing the negative emotions of patients (13), both of which included 20 items. According to each item, a four-level score was adopted according to the frequency of symptoms. The higher the total score, the more serious the degree of anxiety and depression.
4. The incidence of complications, including aneurysm rupture and hemorrhage, cerebrovascular spasm, encephaledema and intracranial infection, were observed and recorded in the two groups.
5. Nutritional status: 5 mL of fasting venous blood was gathered from 2 groups, and the supernatant was obtained after centrifugation. Hemoglobin (Hb), serum albumin (ALB) and prealbumin (PA) levels were measured in the two groups.
6. 5 mL of fasting venous blood was gathered from 2 groups, and the supernatant was obtained after centrifugation. The levels of immunoglobulin A (IgA), immunoglobulin G (IgG) as well as immunoglobulin M (IgM) were detected by immunoturbidimetry.
7. The WHOQOL-BREF was adopted to evaluate the quality of life of patients (14). The scale covered 5 dimensions: physical, psychological, social, and environmental, with a total of 26 items. The total score of each dimension was 100 points, which was proportional to the quality of life.
8. Nursing satisfaction was assessed using the satisfaction questionnaire designed by our hospital with a total score of 100, ≥81 was very satisfied, 60–80 was basically satisfied, and ≤59 was dissatisfied. Satisfaction = (number of very satisfied cases + number of basically satisfied cases)/total cases × 100%.

### Statistical analysis

SPSS 24.0 statistical software was adopted for data analysis. Measurement data were expressed as (x ± s), and t-test was adopted for comparison. Count data were expressed as (*n*, %), and *χ*^2^ test was used for comparison. *P* < 0.05 meant statistical significance.

## Results

### Clinical indexes in 2 groups

**Figure 1:**
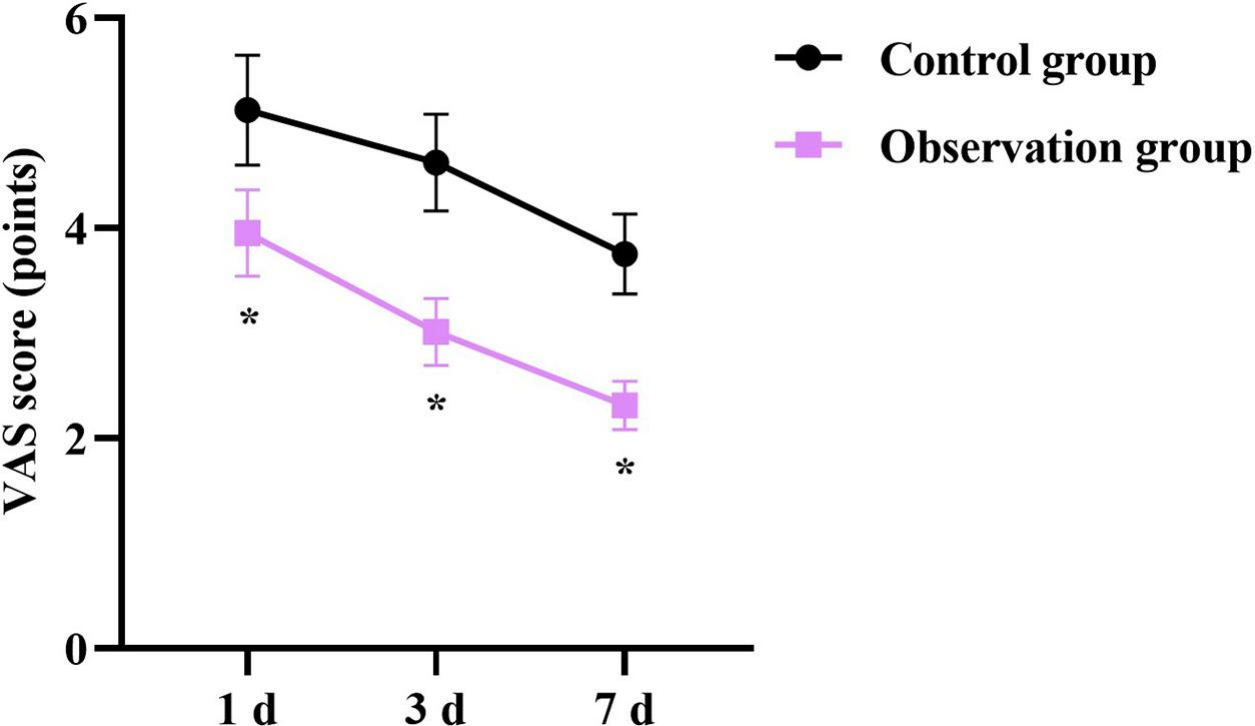
Clinical indexes in 2 groups. *P < 0.05.

In contrast to the CG, the first time of getting out of bed, gastrointestinal function recovery time and length of hospital stay in the OG were shorter (*P* < 0.05, Figure 1).

### Degree of pain in 2 groups

**Figure 2:**
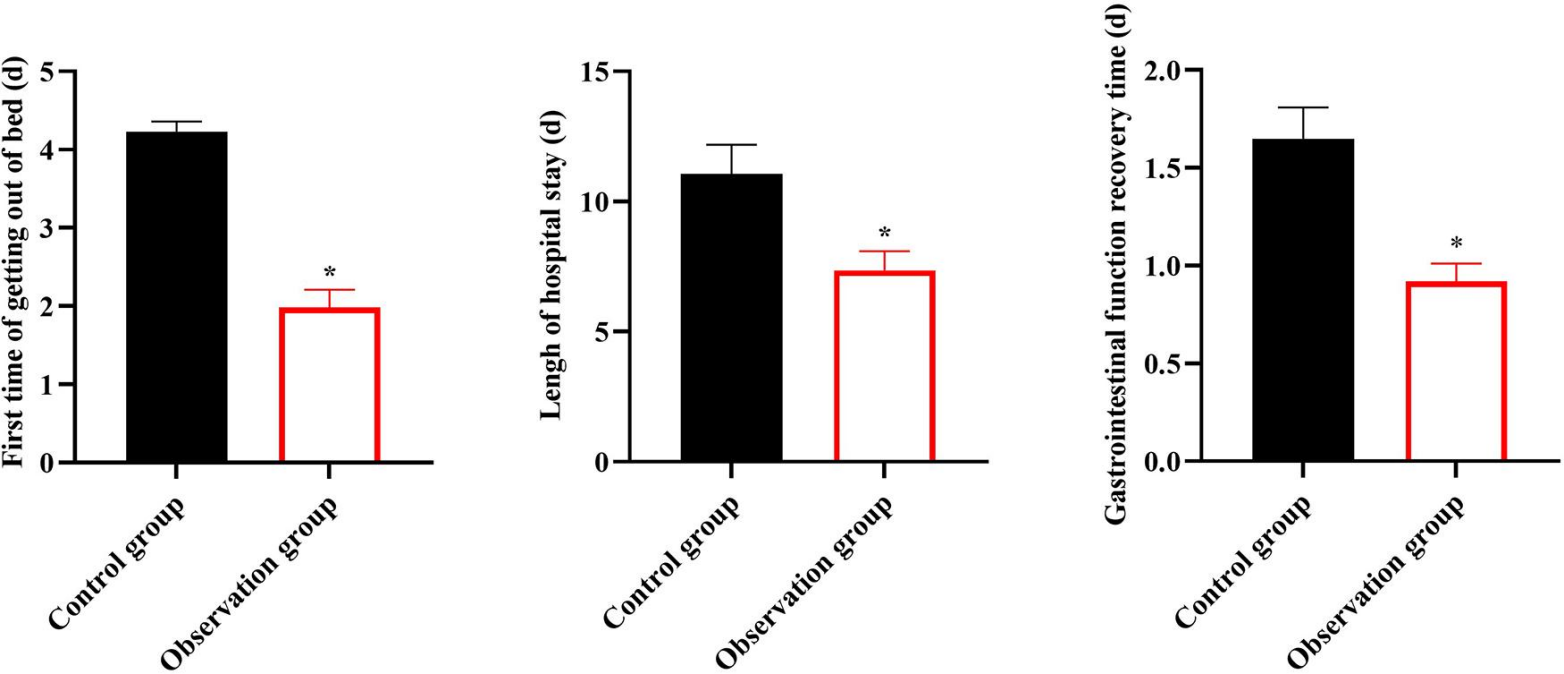
Degree of pain in 2 groups. *p < 0.05.

In contrast to the CG, the VAS scores of the OG at day 1, day 3 and day 7 after surgery were lower (*P* < 0.05, Figure 2).

### Negative emotions of patients in 2 groups

**Figure 3:**
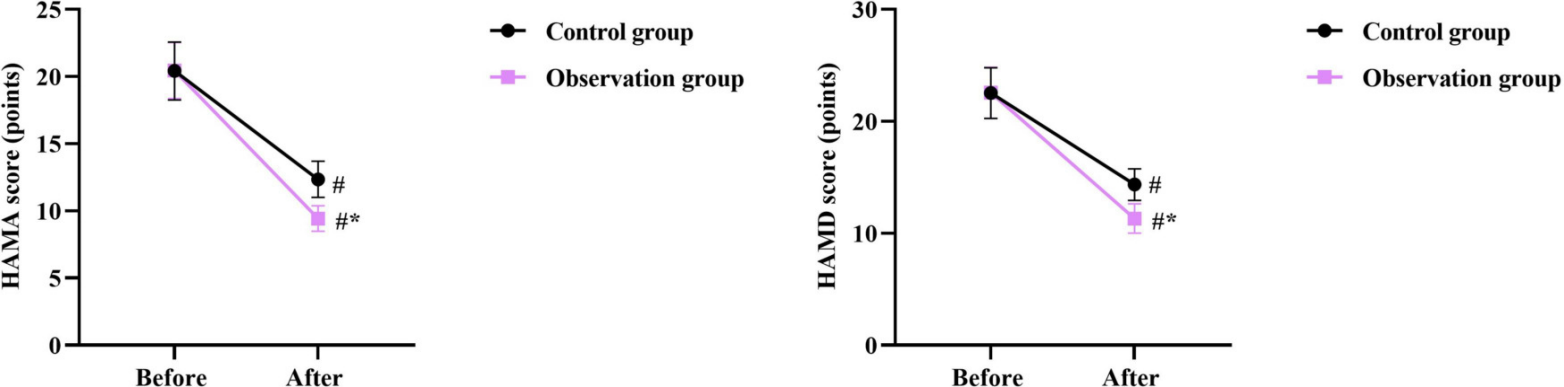
Negative emotions of patients in 2 groups. ^#^*P* < 0.05, in contrast to before intervention, *P < 0.05, in contrast to control group.

Prior to intervention, no differences were seen in HAMA and HAMD scores between the two groups (*P* > 0.05). After intervention, the HAMA and HAMD scores were declined in the two groups, and those in the OG were lower compared with the CG (*P* < 0.05, Figure 3).

### Incidence of complications in 2 groups

In contrast to the CG, the incidence of complications in the OG was lower (*P* < 0.05, Table 2).

**Table 2:** Incidence of complications in 2 groups.

Groups	N	Aneurysm rupture and hemorrhage	Cerebrovascular spasm	Encephaledema	Intracranial infection	Total incidence rate
Control group	69	2	2	3	2	9 (13.04%)
Observation group	69	0	1	1	0	2 (2.90%)
χ^2^						4.840
*P*						0.027

### Nutritional status in 2 groups

**Figure 4:**
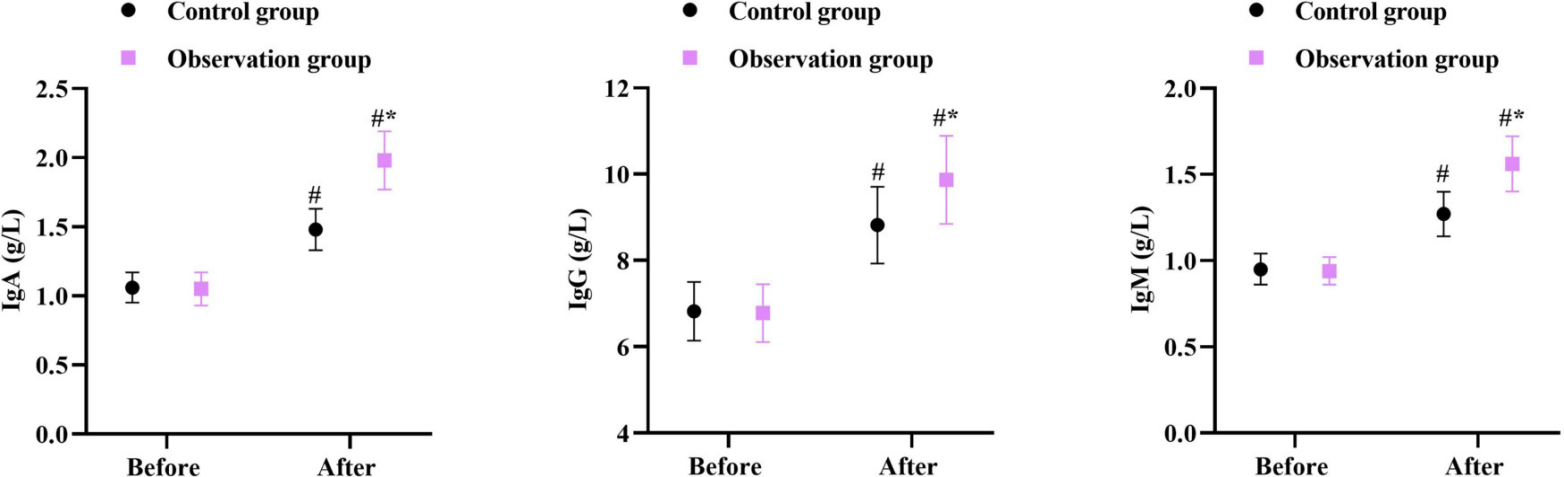
Nutritional status in 2 groups. ^#^*P* < 0.05, in contrast to before intervention, *P < 0.05, in contrast to control group.

Prior to intervention, no differences were seen in Hb, ALB and PA levels between the two groups (*P* > 0.05). After intervention, the Hb, ALB and PA levels were elevated in 2 groups, and those in the OG were higher compared with the CG (*P* < 0.05, Figure 4).

### Immune function in 2 groups

**Figure 5:**
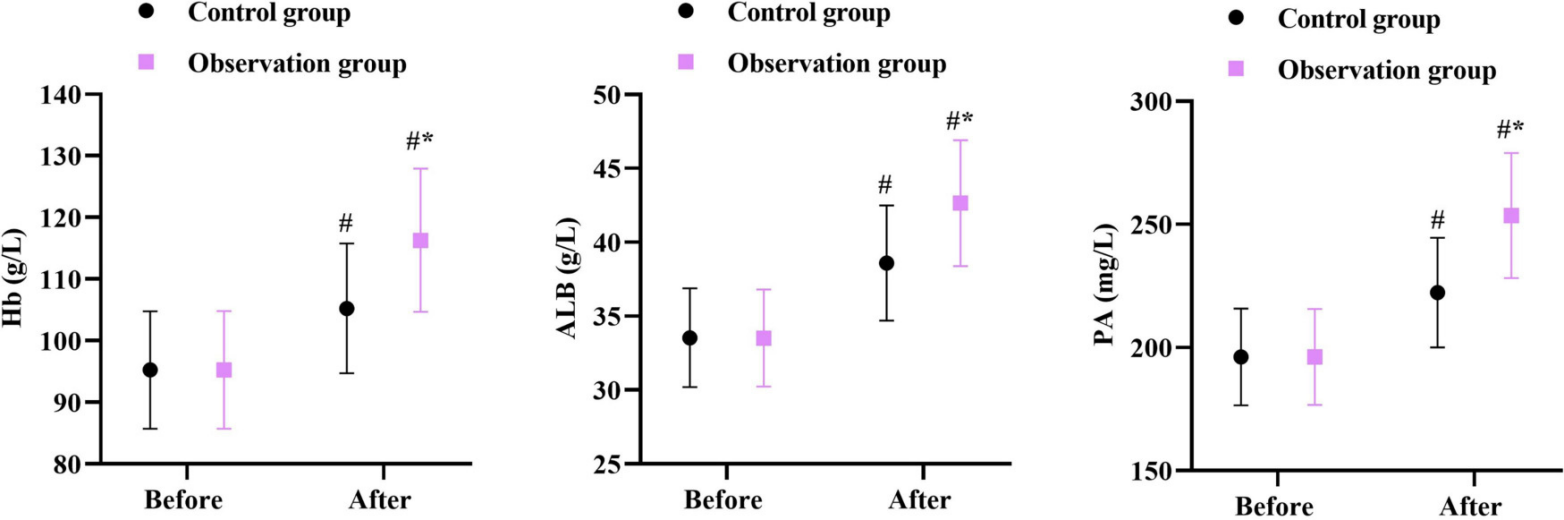
Immune function in 2 groups. ^#^*P* < 0.05, in contrast to before intervention, *P < 0.05, in contrast to control group.

Prior to intervention, no differences were seen in IgA, IgG and IgM levels between the two groups (*P* > 0.05). After intervention, the IgA, IgG and IgM levels were elevated in 2 groups, and those in the OG were higher compared with the CG (*P* < 0.05, Figure 5).

### Quality of life in 2 groups

**Figure 6:**
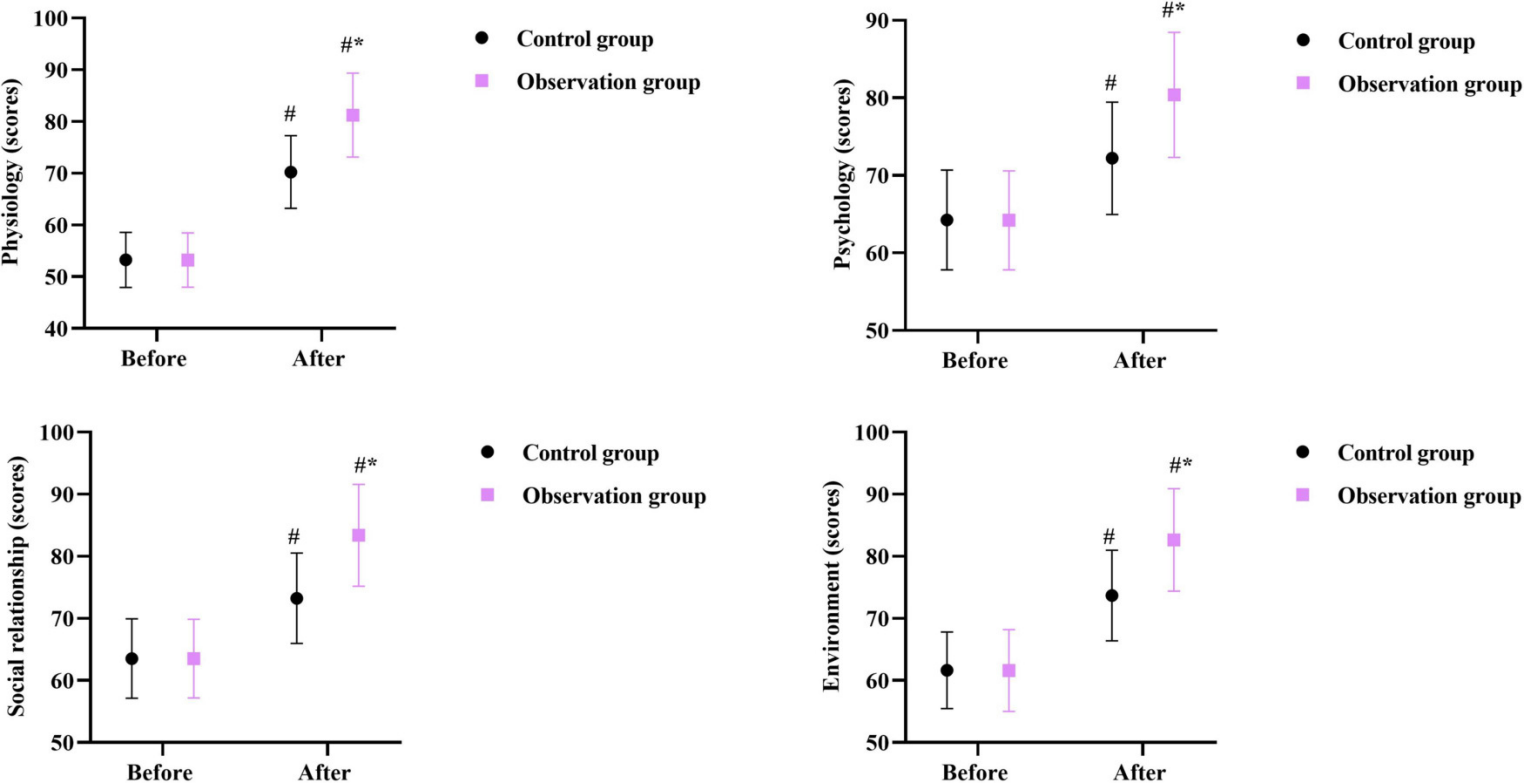
Quality of life in 2 groups. ^#^*P* < 0.05, in contrast to before intervention, *P < 0.05, in contrast to control group.

Prior to intervention, no differences were seen in WHOQOL-BREF scores between the two groups (*P* > 0.05). After intervention, the WHOQOL-BREF scores were elevated in 2 groups, and those in the OG were higher compared with the CG (*P* < 0.05, Figure 6).

### Nursing satisfaction in 2 groups

 Table 3 displayed that in contrast to the CG, the nursing satisfaction of patients in the OG was better (*P* < 0.05).

**Table 3:** Nursing satisfaction in 2 groups.

Groups	N	Very satisfied	Basically satisfied	Dissatisfied	Total satisfaction rate
Control group	69	30	29	10	59 (85.51%)
Observation group	69	38	29	2	67 (97.10%)
χ^2^					5.841
*P*					0.015

## Discussion

There are various causes of intracranial aneurysms, most of which are related to congenital dysplasia of the cerebral artery wall, hypertension, atherosclerosis, and other basic diseases; therefore, the incidence of is high in middle-aged and elderly people (15). The pathogenesis of intracranial aneurysms is complex and has not yet been fully elucidated. Some studies have suggested that it is related to local defects in the arterial wall and abnormal hemodynamics (16). Excessive thinking, forced defecation, and heavy physical labor may lead to the rupture of intracranial aneurysms, and if not treated in a timely manner, often lead to disability or even death of patients (17). Recently, with the continuous improvement of medical technology, interventional embolization has become an ideal means to treat intracranial aneurysms. However, the operation is difficult, and patients are prone to anxiety, fear, and other negative emotions (18). Negative emotions may lead to dysfunction of the nervous system, endocrine system and other functions, leading to increased blood pressure, which can easily cause intracranial aneurysm rupture or postoperative aneurysm secondary rupture, which not only increases the difficulty of surgery, but also seriously affects the prognosis and rehabilitation of patients (19). Therefore, the implementation of effective nursing interventions in the perioperative period of intracranial aneurysm and improvement of patients' psychological status are of great significance to the prognosis of the disease. Fine nursing is a kind of personalized high-quality nursing, which pays attention to the needs of patients on the basis of basic nursing, and provides professional nursing measures from the details to improve the comfort of patients (20).

Patients with intracranial aneurysms are in a state of high decomposition, high metabolism, and negative nitrogen balance, with increased energy demand and accelerated protein renewal (21). Parenteral nutrition can provide the nutrients required by the body; however, long-term total parenteral nutrition can lead to complications such as intestinal mucosa atrophy, intestinal bacterial displacement, and even intestinal sepsis (22). Early enteral nutrition can not only effectively enhance the nutritional status of the body (23) but also activate intestinal nerves and promote the growth of the gastrointestinal mucosa, thereby maintaining the intestinal mucosal barrier, reducing bacterial and toxin displacement, maintaining the normal growth of intestinal flora, and reducing inflammatory response (24).

In our study, the results indicated that, in contrast to the CG, the first time getting out of bed, gastrointestinal function recovery time, and length of hospital stay in the OG were shorter, the VAS scores of the OG at day 1, day 3, and day 7 after surgery were lower, and the incidence of complications in the OG was lower, suggesting that early enteral nutrition combined with fine nursing could promote postoperative recovery, relieve pain, and reduce the incidence of complications in patients with intracranial aneurysms undergoing minimally invasive surgery, which was consistent with previous studies (25, 26).

Our study indicated that after intervention, the HAMA and HAMD scores in the OG were lower than those in the CG, the WHOQOL-BREF scores in the OG were higher than those in the CG, and the nursing satisfaction of the OG was better than that of the CG, suggesting that early enteral nutrition combined with fine nursing could relieve negative emotions, elevate the quality of life, and increase the nursing satisfaction of patients with intracranial aneurysms undergoing minimally invasive surgery. Consistently, Yan et al. indicated that fine nursing could promote the quality of life of patients with severe pneumonia combined with respiratory failure (27).

Moreover, our study indicated that after intervention, the Hb, ALB, and PA levels in the OG were higher than those in the CG, and the IgA, IgG, and IgM levels in the OG were higher than those in the CG, suggesting that early enteral nutrition combined with fine nursing could promote the nutritional status and immune function of patients with intracranial aneurysms undergoing minimally invasive surgery, which was similar to previous reports (28, 29).

However, our study has some limitations. First, this study did not conduct follow-up. Therefore, no in-depth investigation has been carried out regarding the long-term prognosis of the patients, such as their recovery status one year after the surgery or even longer, the risk of recurrence, and the changes in long-term quality of life. Future studies should extend the follow-up period to gain a more comprehensive understanding of the long-term health effects of this intervention on patients. Second, in this study, due to the nature of the nursing intervention, it was difficult to implement blinding for both the nursing staff and the patients. The nursing staff were clearly aware of the nursing plan that the patients received, which might to some extent affect their nursing behaviors and attitudes. At the same time, patients might also experience psychological differences due to knowing which nursing group they were in. This psychological factor might influence their perception and reporting of their own symptoms, thereby affecting the objectivity of the research results. Although we tried to standardize the nursing operations and assessment criteria during the research process, the lack of blinding still might introduce a certain degree of bias. Future research can consider adopting double-blind or single-blind designs to reduce the impact of this bias on the research results. Third, in this study, we failed to fully consider the patient's comorbid conditions and the complexity of the surgery, as well as the complex influence on the research results. Future studies should more thoroughly evaluate the surgical complexity indicators and conduct a more detailed stratified analysis of comorbidities to more accurately assess the effect of early enteral nutrition combined with fine nursing in different patient groups.

## Conclusion

Our study clarifies that early enteral nutrition combined with fine nursing can promote the postoperative recovery, reduce pain, promote nutritional status and immune function, and promote the quality of life of patients with intracranial aneurysm undergoing minimally invasive surgery. The results of this study have laid a foundation for further related research. Subsequent studies can build upon this research to deeply explore the mechanism of action of early enteral nutrition combined with fine nursing, optimize the intervention plan, and explore its application effect in patients with different types of intracranial aneurysms. At the same time, this comprehensive intervention model can also be extended and applied to the postoperative rehabilitation management of other neurosurgical diseases, bringing benefits to more patients.

## Data Availability

The datasets presented in this study can be found in online repositories. The names of the repository/repositories and accession number(s) can be found in the article/Supplementary Material.

## References

[1] Brown RD Jr. Broderick JP. Unruptured intracranial aneurysms: epidemiology, natural history, management options, and familial screening. Lancet Neurol. (2014) 13(4):393–404. 10.1016/S1474-4422(14)70015-8 24646873

[2] Etminan N Rinkel GJ. Unruptured intracranial aneurysms: development, rupture and preventive management. Nat Rev Neurol. (2016) 12(12):699–713. 10.1038/nrneurol.2016.150 27808265

[3] Shi L Yuan Y Guo Y Yu J. Intracranial post-embolization residual or recurrent aneurysms: current management using surgical clipping. Int Neuroradiol. (2016) 22(4):413–9. 10.1177/1591019916647193 PMC4984392 27177873

[4] Liu Y Wang J Lin L Sang C Lin Z Pan Y Clinical study on complications of intracranial ruptured aneurysm embolization by stent-assisted coil. Med Sci Monit. (2018) 24:8115–24. 10.12659/MSM.911773 30419569 PMC6243916

[5] Waili G Amuti S. Influence of humanized nursing on patients with intracranial aneurysm subarachnoid hemorrhage undergoing interventional embolization. Heliyon. (2023) 9(3):e14311. 10.1016/j.heliyon.2023.e14311 36938413 PMC10018550

[6] Yu QN Ji ZH. Fine nursing intervention relieves the clinical symptoms and decreases the adverse events in acute alcoholism patients. Am J Transl Res. (2021) 13(10):11671–9. 34786093 PMC8581946

[7] Berikashvili LB Shestopalov AE Polyakov PA Yakovleva AV Yadgarov MY Kuznetsov IV The neurological metabolic phenotype in prolonged/chronic critical illness: propensity score matched analysis of nutrition and outcomes. Nutrients. (2025) 17(14):2302. 10.3390/nu17142302 40732927 PMC12298954

[8] Surve RM Mishra RK Malla SR Kamath S Chakrabarti DR Kulanthaivelu K Clinical characteristics and outcomes of critically ill neurological patients with COVID-19 infection in neuro-intensive care unit: a retrospective study. Indian J Crit Care Med. (2021) 25(10):1126–32. 10.5005/jp-journals-10071-23989 34916744 PMC8645809

[9] Gao Z Mo X Ge Y Lu Y Yu S. The relationship between elderly nutritional risk index and short-term all-cause mortality in critically ill patients with cerebral injury: a retrospective cohort study from two cohorts. Front Nutr. (2025) 12:1620364. 10.3389/fnut.2025.1620364 40777171 PMC12328167

[10] Peng F Wang H Li J Ma M Jiang X Run H Best evidence summary for prevention and management of enteral feeding intolerance in critically ill patients. J Clin Nurs. (2024) 33(3):781–96. 10.1111/jocn.16934 37994227

[11] Alsharif DJ Alsharif FJ Aljuraiban GS Abulmeaty MMA. Effect of supplemental parenteral nutrition versus enteral nutrition alone on clinical outcomes in critically ill adult patients: a systematic review and meta-analysis of randomized controlled trials. Nutrients. (2020) 12(10):2968. 10.3390/nu12102968 32998412 PMC7601814

[12] Tian X Song W Xia G Tan C Yin J. Research progress of the effect of enteral nutrition on intestinal microecology in neurocritical ill patients. Zhonghua Wei Zhong Bing Ji Jiu Yi Xue. (2021) 33(11):1393–6. 10.3760/cma.j.cn121430-20210424-00607 34980317

[13] Meng J Du J Diao X Zou Y. Effects of an evidence-based nursing intervention on prevention of anxiety and depression in the postpartum period. Stress Health. (2022) 38(3):435–42. 10.1002/smi.3104 34633141

[14] Skevington SM Lotfy M O'Connell KA. The world health organization’s WHOQOL-BREF quality of life assessment: psychometric properties and results of the international field trial. A report from the WHOQOL group. Quality of Life Res. (2004) 13(2):299–310. 10.1023/B:QURE.0000018486.91360.00 15085902

[15] Micieli JA Newman NJ Barrow DL Biousse V. Intracranial aneurysms of neuro-ophthalmologic relevance. J Neuro-ophthalmol. (2017) 37(4):421–39. 10.1097/WNO.0000000000000515 28665866

[16] Gasparotti R Liserre R. Intracranial aneurysms. Eur Radiol. (2005) 15(3):441–7. 10.1007/s00330-004-2614-8 15678323

[17] Gilbert ME Sergott RC. Intracranial aneurysms. Curr Opin Ophthalmol. (2006) 17(6):513–8. 10.1097/ICU.0b013e328010a1e7 17065918

[18] Li TF Shui SF Han XW Yan L Ma J Guo D. One-Stage endovascular embolization for multiple intracranial aneurysms. Turk Neurosurg. (2018) 28(1):43–7. 10.5137/1019-5149.JTN.18186-16.1 27593847

[19] van der Schaaf IC Wermer MJ Velthuis BK Buskens E Bossuyt PM Rinkel GJ. Psychosocial impact of finding small aneurysms that are left untreated in patients previously operated on for ruptured aneurysms. J Neurol Neurosurg Psychiatr. (2006) 77(6):748–52. 10.1136/jnnp.2005.079194 PMC2077475 16705198

[20] Cao M. Fine nursing model combined with psychological intervention on patients after eyeball enucleation due to ocular trauma. Am J Transl Res. (2021) 13(6):7071–6. 34306466 PMC8290637

[21] Xu Z Rui YN Hagan JP Kim DH. Intracranial aneurysms: pathology, genetics, and molecular mechanisms. NeuroMol Med. (2019) 21(4):325–43. 10.1007/s12017-019-08537-7 PMC6829066 31055715

[22] Plogsted S Adams SC Allen K Cober MP Greaves J Mogensen KM Parenteral nutrition amino acids product shortage considerations. Nutr Clin Pract. (2016) 31(4):560–1. 10.1177/0884533616653834 27296811

[23] Reintam Blaser A Starkopf J Alhazzani W Berger MM Casaer MP Deane AM Early enteral nutrition in critically ill patients: eSICM clinical practice guidelines. Intensive Care Med. (2017) 43(3):380–98. 10.1007/s00134-016-4665-0 28168570 PMC5323492

[24] Moon SJ Ko RE Park CM Suh GY Hwang J Chung CR. The effectiveness of early enteral nutrition on clinical outcomes in critically ill sepsis patients: a systematic review. Nutrients. (2023) 15(14):3201. 10.3390/nu15143201 37513620 PMC10383540

[25] Stannard D. Early enteral nutrition within 24 hours of lower gastrointestinal surgery versus later commencement for length of hospital stay and postoperative complications. J Perianesth Nurs. (2020) 35(5):541–2. 10.1016/j.jopan.2020.07.003 33010850

[26] Hu Z Zou D Fu X Zhou W. Effect of fine nursing with dietary intervention on pain level of patients with advanced lung cancer. Am J Transl Res. (2023) 15(4):2738–46. 37193141 PMC10182513

[27] Yan X Zhang H Pang H Liu C. Effect of fine nursing intervention on therapeutic effect and quality of life of patients with severe pneumonia complicated with respiratory failure. Minerva Med. (2021) 112(5):758. 10.23736/S0026-4806.21.07718-1 34542946

[28] Sun JK Mu XW Li WQ Tong ZH Li J Zheng SY. Effects of early enteral nutrition on immune function of severe acute pancreatitis patients. World J Gastroenterol. (2013) 19(6):917–22. 10.3748/wjg.v19.i6.917 23431120 PMC3574890

[29] Ma BQ Chen SY Jiang ZB Wu B He Y Wang XX Effect of postoperative early enteral nutrition on clinical outcomes and immune function of cholangiocarcinoma patients with malignant obstructive jaundice. World J Gastroenterol. (2020) 26(46):7405–15. 10.3748/wjg.v26.i46.7405 33362392 PMC7739166

